# Essentials of Diseases of the Skin, Including the Syphilodermata, Arranged in the Form of Questions and Answers

**Published:** 1900-11

**Authors:** 


ESSENTIALS OF DISEASES OF THE SKIN, including the Syphilodermata, arranged in the form of questions and answers. Prepared especially for students of medicine. By Henry W. Stelwagon, M. D., Ph. D., Clinical Professor of Dermatology in the Jefferson Medical College, Etc., Philadelphia. Fourth edition, thoroughly revised. Illustrated. Pages, 276; price, $1.00. Philadelphia: W. B. Saunders, 925 Walnut Street. 1899.

This little book belongs to the series of Saunders' Question Compends, No. 11. This edition shows the work in its improved state. All the necessary changes to bring the book up to date have been made. This work meets all the demands for which it was gotten out, and for a "crammer" there is none better.

